# Sleep complaints associates with anxiety in individuals with Subjective Cognitive Decline from a South‐Brazilian cohort

**DOI:** 10.1002/alz.093098

**Published:** 2025-01-03

**Authors:** Gabriela Raquel Paz Rivas, Victória Tizeli Souza, Simone Sieben da Mota, Bruno De Oliveira De Marchi, Guilherme Da Silva Carvalho, Haniel Bispo De Souza, Lucas Bastos Beltrami, Rhaná Carolina Santos, Sarah Vitoria Bristot Carnevalli, Ana Letícia Amorim de Albuquerque, Leonardo Martins de Paula, Manuella Edler Zandoná Giordani, Wyllians Vendramini Borelli, Giovanna Carello‐Collar, Marcia L. Fagundes Chaves, Eduardo R. Zimmer, Raphael Machado Castilhos

**Affiliations:** ^1^ Universidade Federal do Rio Grande do Sul, Porto Alegre, RS Brazil; ^2^ Hospital de Clínicas de Porto Alegre, Porto Alegre, Rio Grande do Sul Brazil; ^3^ Universidade do Vale do Rio dos Sinos, São Leopoldo, Rio Grande do Sul Brazil; ^4^ Universidade Federal de Santa Catarina, Florianópolis Brazil; ^5^ Memory Center, Hospital Moinhos de Vento, Porto Alegre, RS Brazil

## Abstract

**Background:**

Subjective cognitive decline (SCD) is defined by the presence of cognitive complaints in cognitively unimpaired (CU) individuals. Despite being part of the continuum of a neurodegenerative disease, the cause of the cognitive complaint is quite heterogeneous. Sleep disturbances may be associated with cognitive complaints and even objective cognitive decline. Therefore, our aim is to analyse sleep complaints in patients with SCD evaluated in a south‐Brazilian SCD (BRASCODE) cohort.

**Method:**

We included CU individuals aged > 65 years‐old with cognitive complaints from the BRASCODE cohort. Exclusion criteria were uncontrolled psychiatric/clinical illness, cerebrovascular disease, and the use of psychotropic drugs. We applied the Memory Complaint Scale (MCS) (in both participant and informant), Geriatric Anxiety Inventory (GAI) and Geriatric Depression Scale (GDS), Pittsburgh Sleep Quality Index (PSQI), Mini Mental State Examination (MMSE), Mild Behavioral Impairment (MBI) and Neuropsychiatric Inventory (NPI) questionnaires. For this analysis we used the data collected in the 12‐month follow‐up only.

**Result:**

Forty‐seven participants completed the 12‐month evaluation between March, 2022 and November, 2023. Of these, 74.5% (n = 35) were women, median age of 71 (69‐74.5) years‐old and 16 (11‐18) years of formal education. Their median interquartile range (IQR) on SCD‐patient and SCD‐informant scale was 8 (6‐9.5) and 4 (2‐6), respectively. The remaining scale’s ‐median and IQR scores‐ were the following: PSQI, 7 (4‐9); GAI, 6 (3‐9); GDS 2 (1‐4), MMSE, 29 (28‐30); NIP,1(0‐4); MBI‐patient, 3 (0.75‐6); and MBI‐informant, 3 (1‐9). The analysis showed that the PSQI scale only had correlation with GAI (p‐value = 0.02841, rho = 0.3466809). There was no association with the MCS‐participant and MCS‐informant scales, nor with MMSE, age, education and behavior scales, MBI or NPI.

**Conclusion:**

This result obtained for the PSQI score suggests that poor sleep quality in SCD individuals from a south‐Brazilian cohort may be associated with anxiety symptoms. Larger sample may provide additional information about the relationship of cognitive, anxiety and sleep complaints.

